# Correlation Between Chronic Periodontitis and Lung Cancer: A Systematic Review With Meta-Analysis

**DOI:** 10.7759/cureus.36476

**Published:** 2023-03-21

**Authors:** Umesh P Verma, Pooja Singh, Ajay K Verma

**Affiliations:** 1 Department of Periodontology, King George's Medical University, Lucknow, IND; 2 Department of Pulmonary Medicine, King George's Medical University, Lucknow, IND

**Keywords:** signaling pathway, systemic inflammation, meta-analysis, lung cancer, chronic periodontitis

## Abstract

Periodontal disease is associated with many systemic diseases, such as cardiovascular diseases, atherosclerosis, diabetes mellitus, stroke, and pulmonary diseases. Interestingly, recent literature suggests that periodontal disease might be a risk factor for various cancers such as lung, colon, oesophageal, head, and neck cancers. However, the precise mechanistic link is lacking. Hence, in this meta-analysis, we aimed to investigate the correlation between periodontal disease and lung cancer in periodontally diseased patients. Data were searched for relevant studies from 2010 to 2022. We correlated periodontal disease and lung cancer based on adjusted ORs/HRs and associated CIs. I2 statistic was used to assess statistical heterogeneity. Publication bias was analyzed by visually inspecting the symmetry of the funnel plot and Egger's test. The study is registered in the International Prospective Register of Systematic Reviews (PROSPERO; registration no: CRD42023390819). A total of 194,850 participants from observational studies (two case-control studies and five cohort studies) were incorporated for the current analysis. The meta-analysis of included studies showed an overall effect size (risk ratio) of the periodontal disease group with respect to the non-periodontal disease group for lung neoplasm to be 1.41 (95% CI: 1.32-1.52). The value was more than 1, indicating that the periodontal disease group had a relatively higher lung cancer prevalence than the non-periodontal disease group. Further, the overall risk ratio was found to be statistically significant (p<0.00001). Moreover, the funnel plot suggested some degree of publication bias. Evidence in our study implicated that there is an increased risk of occurrence of lung cancer in chronic periodontitis patients.

## Introduction and background

Cancer is a significant etiologic factor in lowering life expectancy and one of the main reasons for death worldwide [1]. In 2020, there was a worldwide estimate of approximately 19.3 million new carcinoma cases and their associated deaths, counting to 10 million. Lung neoplasm is the most frequently occurring neoplasm, ranked second worldwide, and a leading reason of death due to cancer. Chronic periodontitis is a widespread oral health issue in public across the world and is ranked at sixth position. Around 10.8% of the world population is affected by chronic periodontitis [2]. Periodontal disease is a chronic inflammation of the gingiva and supporting structure around the tooth consisting of cementum, alveolar bone, and periodontal ligament. Initially, it starts with gingivitis which is caused by polymicrobial dental plaque. The chance of developing specific cancers, such as those of the brain, lungs, female reproductive system, and digestive tract, was much lower when periodontal disease was treated. This association between periodontal disease and cancer risk indicates the possibility of a positive link between them [3]. However, the exact mechanism underpinning this association is still debatable. However, various mechanisms have been proposed suggesting the risk of cancer development in chronic periodontitis patients, including systemic inflammation [4]. Periodontal infection can lead to the spreading of inflammation far off the oral cavity and results in an increase in inflammatory markers in systemic circulation [5]. Moreover, sites of pre-cancerous and cancerous lesions have been shown to harbor periodontal pathogens and favor a pro-carcinogenic milieu. Compelling evidence has suggested that periodontally diseased patients are more likely to have pulmonary diseases such as chronic obstructive pulmonary disease (COPD) and pneumonia. Moreover, studies have also shown that pulmonary disease, never-smoking sufferers, were more susceptible to future risk for developing lung cancer. A close anatomical approximation between the lungs and oral cavity could explain a biologically plausible reason for the relationship between lung cancer and periodontal disease [6]. As we move from the oral cavity to the lungs, the diversifying microbiome narrows down comparatively. While certain studies have documented that lung cancer risk is increased in individuals with poor oral health, the exact causal relationship is debatable. Although a couple of meta-analyses have been conducted to answer a similar research question, we were tempted to conduct this updated meta-analysis to present the latest evidence due to some recent observational studies done in this sphere.

## Review

Methodology

Study Selection

**Figure 1:**
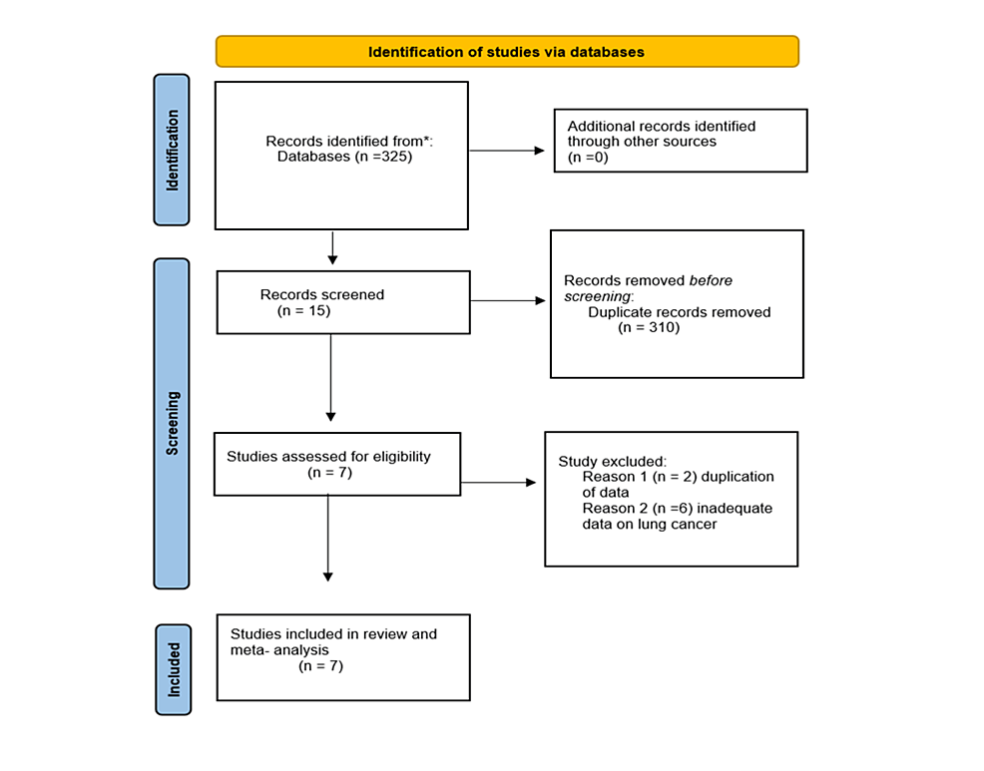
PRISMA flow diagram for study selection for meta-analysis. PRISMA: Preferred Reporting Items for Systematic Reviews and Meta-Analyses.

This systematic review and meta-analysis were done to determine the risk of lung cancer in chronic periodontitis patients. Three hundred twenty-five studies were initially obtained from the literature search (PubMed, Science Direct, Medline, Cochrane, and Embase databases). Initially, 15 studies were included based on the titles and abstracts of the articles. Out of these, seven studies were assessed for our meta-analysis, comprising five cohort studies and two case-control studies. The criteria for study selection were based on Preferred Reporting Items for Systematic Reviews and Meta-Analyses (PRISMA) (Figure 1) [7].

For cohort and case-control studies, the Newcastle-Ottawa Scale (NOS) was used to assess the method's quality, including participant selection, comparability, result assessment, and exposure (Table 1) [8].

**Table 1:** Newcastle-Ottawa quality assessment scale.

Selection	Cohort studies					
		Nwizu NN et al. [9]	Arora M et al. [10]	Mai X et al. [11]	Michaud DS et al. [12]	Michaud DS et al. [13]
	Representativeness of the exposed cohort	√	√	√	√	√
	Selection of the nonexposed cohort	√	√	√	√	√
	Ascertainment of exposure		√			√
	Demonstration that outcome of interest was not present at start of study	√	√	√	√	√
	Case-control study					
		Chrysanthakopoulos NA [14]	Yoon HS et al. [15]			
	Is the case definition adequate?	√	√			
	Representativeness of the cases	√	√			
	Selection of controls		√			
	Definition of controls	√	√			

Search Strategy

We explored the observational studies demonstrating a correlation between lung cancer and periodontal disease through a search engine from January 2010 to December 2022. All related studies in English were identified with the following search terms "periodontal disease" OR "periodontitis" OR "probing pocket depth" OR "pyorrhoea" AND "lung cancer" OR "neoplasm of lung."

Eligibility Criteria

Screening of the title and abstract of all studies was done. The complete text of seven likely pertinent articles [9-15] was evaluated further. In the meta-analysis, studies fulfilling the following inclusion criteria were considered: (A) original observational studies; (B) periodontal disease as a topic of concern; (C) incidence and mortality of lung cancer in terms of outcome; (D) study incorporating lung cancer correlated with any estimate of periodontal disease, 95% CIs, or HRs, ORs, RRs; and (E) articles published in English language.

Data Collection Process

The risk of bias/quality assessment of all the articles included was done by two reviewers (XYZ and MYX) who assessed the studies based on inclusion and exclusion criteria. The author's surname, study duration, year of publication, sample size, study design, population characteristics, exposure, outcome, and adjusted data of HRs/ORs/RRs and 95% CI were assessed. Information based on the above criteria was discussed between the authors for any discrepancies.

Data Analysis

RevMan (version 5), a Cochrane software, was used for preparing the review and performing a meta-analysis of our data. The adjusted ORs/HRs and their associated CIs were used as a measure to correlate between lung cancer and periodontal disease, and data was extracted from included studies. The risk ratio of the periodontal disease group with respect to the non-disease group was used to measure the dichotomous outcomes and effect size, both with 95% CI. I2 statistics were used to determine the heterogeneity of the data across studies (Table 2). A fixed effect model was utilized for meta-analysis.

**Table 2:** Sensitivity and subgroup analysis.

		No. of studies				Heterogeneity			Model	Meta-analysis							
						I^2^(%)	P-value			HR			95% CI			P-value	
Subgroup analyses																	
Study design																	
Prospective cohort			5		0.84		<0.001	Fixed-effect			1.61			1.44-1.80			<0.001
Retrospective cohort			2		0		0.86	Fixed-effect			1.31			1.19-1.43			<0.001
Country of origin																	
Asia	1			NA			NA		NA			1.41			0.84-2.36	0.19	
Europe	1			NA			NA		NA			1.31			1.19-1.44	<0.001	
USA	5			0.84			<0.001		Fixed-effect			1.57			1.41-1.75	<0.001	
Adjusted covariates																	
Smoking		2				0	0.35		Fixed-effect	1.32			1.20-1.44			<0.001	
Smoking, alcohol		2				0.9	0.002		Fixed-effect	1.91			1.62-2.25			<0.001	
Smoking, alcohol, and diabetes		2				0	0.87		Fixed-effect	1.35			1.15-1.59			<0.001	
Gender																	
Female and Male		5				0	0.65		Fixed-effect	1.33			1.22-1.45			<0.001	
Female		2				0.95	<0.001		Fixed-effect	1.62			1.43-1.84			<0.001	
Sensitivity analyses																	
Study with longest follow-up duration omitted		6				0.85	<0.001		Fixed-effect	1.41			1.32-1.52			<0.001	
Study with shortest follow-up duration omitted		6				0	0.79		Fixed-effect	1.34			1.24-1.44			<0.001	
Co-twin study excluded		6				0.85	<0.001		Fixed-effect	1.41			1.32-1.52			<0.001	
Excluded study without adjustment of smoking status		6				0.85	<0.001		Fixed-effect	1.41			1.31-1.51			<0.01	

Results 

Study Characteristics

Studies published [9-19] from 2010 to 2022 were included in our review. These studies were from USA, Greece, Taiwan, and Sweden (Table 3).

**Table 3:** Baseline characteristics of the studies. * WHI-OS: Women's Health Initiative Observational Study; † HPFS: The Health Professionals Follow-Up Study; ‡ ARIC: Atherosclerosis Risk in Communities.

Author	Study design	Year	Source of population	Study duration	Study outcome
Nwizu NN et al. [9]	Cohort study	2017	USA (WHI-OS)*	1999-2013	Cancer incidence
Arora M et al. [10]	Cohort study	2010	Sweden	1963-2004	Cancer mortality
Mai X et al. [11]	Cohort study	2016	USA (Buffalo OsteoPerio Study)	1997-2014	Cancer incidence
Michaud DS et al. [12]	Cohort study	2016	USA (HPFS)†	1986-2012	Cancer incidence
Michaud DS et al. [13]	Cohort study	2018	USA (ARIC)‡	1987-2012	Cancer incidence
Chrysanthakopoulos NA [14]	Case-control	2016	Greece (Private dental and medical practice)	-	Cancer incidence
Yoon HS et al. [15]	Nested case-control study	2019	USA	2008-2015	Cancer incidence
Chung PC et al. [16]	Cohort study	2020	Taiwan	2005-2012	Cancer mortality
Hujoel PP et al. [17]	Cohort study	2003	USA	1971-1992	Cancer mortality
Michaud DS et al. [18]	Cohort study	2008	USA	1986-2004	Cancer incidence
Mai X et al. [19]	Cohort study	2014	USA (WHI-OS)*	1993-2010	Cancer incidence

Meta-analysis

The meta-analysis of selected studies showed that the overall effect size (risk ratio) of the periodontal disease group with respect to the non-periodontal disease group for lung neoplasm was 1.41 (95% CI: 1.32-1.52) (Table 4) [9-15]. The value was more than 1, which showed that the periodontal disease group had a relatively higher incidence of lung cancer cases than the non-periodontal disease group. Further, the overall risk ratio came out to be statistically significant (p<0.00001). It is noteworthy that the included prospective studies estimated risk ratio by HR while case-control studies estimated it by OR. Heterogeneity: Chi² = 33.90, df = 6 (P < 0.00001); I² = 82%. Overall effect test: Z = 9.60 (P < 0.00001).

**Table 4:** Meta-analysis of lung cancer with association of periodontal disease.

Author		Year	Sample size	Case (Lung Cancer)		Control		Weight	Risk ratio (95% CI)
				Event	Total	Event	Total		
Nwizu NN et al. [9]		2017	5970	70	152	1150	5818	7.90%	2.33 [1.95, 2.79]
Arora M et al. [10]		2010	12817	14	225	556	12592	2.60%	1.41 [0.84, 2.36]
Mai X et al. [11]		2016	2015	127	403	377	1612	20.40%	1.35 [1.14, 1.60]
Michaud DS et al. [12]		2016	200	18	64	20	136	1.70%	1.91 [1.09, 3.36]
Michaud DS et al. [13]		2018	1252	15	17	760	1235	2.80%	1.43 [1.20, 1.72]
Chrysanthakopoulos NA [14]		2016	65869	290	855	16809	65014	59.10%	1.31 [1.19, 1.44]
Yoon HS et al. [15]		2019	19933	25	109	3580	19824	5.30%	1.27 [0.90, 1.79]
	Total (95% CI)			559	1825	23252	106231	100.00%	1.41 [1.32, 1.52]

**Figure 2:**
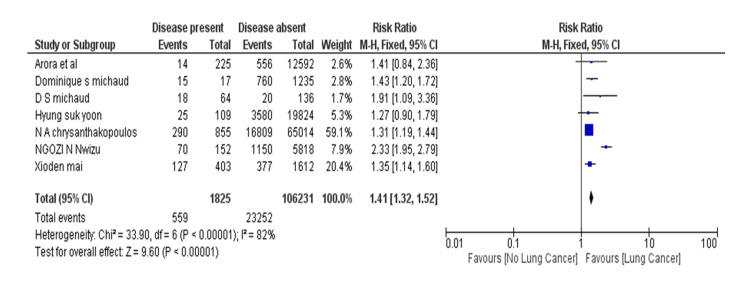
Forest plot of periodontal disease and risk of lung cancer. Source: Nwizu NN et al. [9], Arora M et al. [10], Mai X et al. [11], Michaud DS et al. [12], Michaud DS et al. [13], Chrysanthakopoulos NA [14], Yoon HS et al. [15] * Horizontal lines depicting 95% CIs; † Vertical line is a line of no effect; The right side of vertical line favours the incidence of lung cancer in periodontitis patients; ‡ Black square describing RR or OR of individual study; § Black square size is showing weightage of each study in a meta-analysis; || Diamond is showing the overall treatment effect and center of it is showing the combined treatment effect. As diamond is on the right side of vertical line which is showing the higher chances of lung cancer in periodontitis patient and difference between two groups is statistically significant.

The forest plot point estimate of each study in the graph and the CI have been shown in Figure 2. The diamond shape figure in the forest plot is showing combined point estimate (OR/RR) and CI of the meta-analysis of included studies. The result obtained from statistical analysis of all studies concluded that the periodontal disease group has comparatively higher chances of having lung cancer than the non-periodontal disease group. It was found in our analysis that the overall risk ratio is statistically significant (p<0.00001).

**Figure 3:**
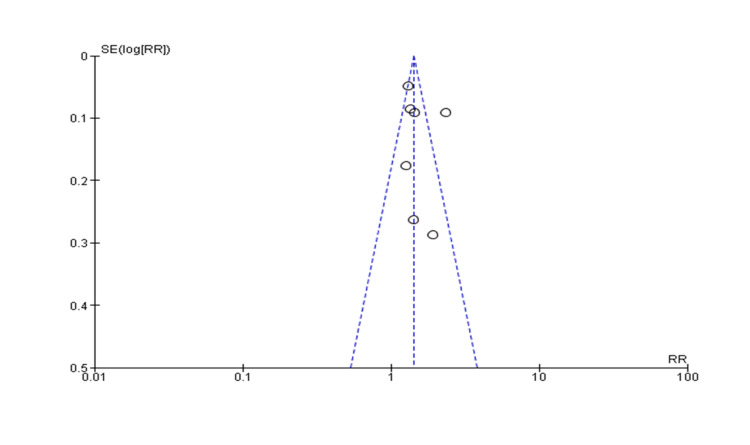
The funnel plot of the included observational studies. The plot demonstrates the study's results (X-axis) and precision (Y-axis). Risk ratios (RRs) and precision are the standard error of the RR shown in the funnel plot. Each dot of the plot represents a separate study. The solid middle line indicates the overall effect of the meta-analysis.

A funnel plot was used for publication bias estimation (Figure 3). In this method, the intervention effect was assessed by scatter plot to estimate from individual studies against some measure of each study's size or precision. In the horizontal scale, we plotted the effect estimates while the measure of study size was plotted over the vertical axis. P<0.05 was taken as significant. It is noteworthy to mention that the included prospective studies estimated risk ratio by the HR while case control studies estimated it by OR. Heterogeneity: Chi² = 33.90, df = 6 (P < 0.00001); I² = 82%. Overall effect test: Z = 9.60 (P < 0.00001).

Discussion

In our meta-analysis, it was found that there is a risk of having lung cancer in chronic periodontitis patients in comparison to non-periodontitis patients. The study showed that the presence of periodontal disease elevates the risk of having carcinoma (HR: 1.14; 95% CI: 1.08-1.20) in older women, irrespective of smoking, during 8.32 years of mean follow-up [9]. The study described the relationship between baseline periodontal disease and carcinoma incidence in Swedish twins. After covariables adjustment, there was an association between baseline periodontal disease and a greater risk of developing neoplasms ranging from 15% for a total neoplasm to 120% for corpus uterine neoplasm. Arora M et al. explained in their study that dizygotic twins with baseline periodontal disease showed an elevated 50% risk for total cancer (HR: 1.50; 95% CI: 1.04-2.17), but in monozygotic twins, this correlation was distinctly attenuated (HR: 1.07; 95% CI: 0.63-1.81) [10]. In another study which was conducted in postmenopausal women, a marginal correlation was found between periodontal pathogens such as Prevotella intermedia, Fusobacterium nucleatum, Campylobacter rectus (orange-colored complex) and risk of having lung carcinoma (HR: 3.02; 95% CI: 0.98-9.29) and total carcinoma risk (HR: 1.35; 95% CI: 1.00-1.84) [11]. Cigarette smoking is a common risk factor for periodontitis and pulmonary cancer. In a study, there was a 2.5 times elevated risk observed for cancers related to smoking (lung, oropharyngeal, bladder, oesophageal, stomach, kidney, and liver carcinoma (HR: 1.33; 95% CI: 1.07-1.65) in men associated with advanced periodontitis (HR: 2.57; 95% CI: 1.56-4.21) in comparison to men who did not have advanced periodontitis [12]. The study demonstrated the relationship between the increased risk of pulmonary carcinoma and periodontal disease in their Atherosclerosis Risk in Communities (ARIC) study through evaluation of dental examination. A significantly increased risk of carcinoma (HR: 1.24; 95% CI: 1.07-1.44) was found for severe periodontitis (>30% of sites having attachment loss of >3 mm) compared with no/mild periodontitis (3 mm), after adjusting smoking and other related factors. A powerful relation was seen for lung cancer (HR: 2.33; 95% CI: 1.51 -3.60) [13]. Another study found an association between indices related to periodontal disease and lung cancer. Clinical attachment loss (95% CI: 1.30-9.47; OR: 3.51) probing pocket depth (95% CI: 1.05-7.06; OR: 2.72) bleeding while probing (95% CI: 0.98-3.81; OR: 1.93,) were associated with an increase in the incidence of lung cancer. Out of all, probing pocket depth was statistically significantly related to lung cancer [14]. It was observed in a study that tooth loss has an association with an elevated risk of developing lung cancer. This was noticed among African Americans (OR: 1.56; 95% CI: 1.05-2.31) and those who smoked heavily (OR: 2.05; 95% CI: 1.38-3.05) and explained the role of poorly maintained oral health in the elevation of carcinoma risk, among a population of European and African Americans in the Southeastern US having low income [15].

The study found conflicting evidence for a link between periodontal disease and mortality from all causes, all cancers, and specific cancers like lung cancer. Risk factors for mortality were being a man, having less education, and smoking [16]. Apart from adjusting for established lung cancer risk factors, associations between periodontitis and lung cancer mortality can be found [17]. The relationship between periodontal disease and overall cancer risk was modest but substantial, and it persisted in never-smokers [18].In postmenopausal non-smokers, periodontal disease was not independently linked to lung cancer. However, the risk of lung cancer from smoking and PD together was higher than would be anticipated from the combined effects of each factor alone [19]. 

Periodontal disease is an inflammatory condition which is caused by a periodontal pathogen and often accompanied by the elevated level of biomarkers like interleukins (IL-1β, IL-6), interferons (IFN-ɣ) and C-reactive protein (CRP) [20-22]. It is well explained in various studies that these elevated inflammatory mediators are involved with the development of lung cancer risk [23-25]. It has been noted that oral bacteria can create disturbance in the health of human beings by releasing toxins which can lead to disturbance in cell physiology [26]. The microorganisms related to the oral cavity is the main source of bacteria in the lungs and study has shown that oral bacteria can increase the risk of lung cancer [27].There were higher salivary levels of Capnocytophaga and Veillonella in pulmonary cancer patients, which might be considered as possible biomarkers for early disease detection [28].In light of the above-discussed studies, the public should be taught about the importance of maintaining their dentition in a healthy state and the potential association of periodontal disease with lung cancer should not be ruled out. Although, the exact pathogenic mechanism behind the association between periodontitis and lung cancer remains unclear and warrants more research in the field of molecular biology.

Limitations of the study

There are certain limitations in our meta-analysis. Firstly, studies that are included are few and the study was conducted in different regions of the world so there can be a variation in the incidence of lung cancer in periodontitis patients. Secondly, there are some confounder factors such as smoking, diabetes, and alcohol which should always be kept in mind for proper assessment of the relationship between periodontitis and lung cancer. Further treatment modalities should be incorporated to predict the reduced risk of cancer in a chronic periodontitis-treated patient. Additionally, there was a lack of data regarding the specific association between clinical parameters of periodontitis and specific histological types of lung cancer. Furthermore, there was a major difference in reporting clinical parameters methods for chronic periodontitis assessment and lung cancer association between included studies which might create a source of heterogeneity.

## Conclusions

The results of our meta-analysis summarized and suggested a possible correlation between periodontitis and lung cancer risk. Chronic periodontitis is an inflammatory condition of the periodontium. It can elevate the risk of lung cancer in susceptible individuals by creating an inflammatory milieu. Preventive and therapeutic measures are well known for periodontal diseases and should be implemented, keeping in mind improving the local as well as overall systemic health of the patient. However, to further support this association between the two conditions, large-scale studies are required with adequate control/adjustment of confounding factors for a more conclusive result.
